# *Saprochaete clavata* (*Geotrichum clavatum*) septicemia in a patient with multiple myeloma; An emerging case from Southeastern Turkey

**DOI:** 10.18502/cmm.6.4.5440

**Published:** 2020-12

**Authors:** Handan Kangül, Nida Özcan, Nurullah Uzuner, Mahmut Mete, Ufuk Mert Erginer

**Affiliations:** 1 Department of Medical Microbiology, Faculty of Medicine, Dicle University, Diyarbakır, Turkey; 2 Department of Internal Medicine, Faculty of Medicine, Dicle University, Diyarbakır, Turkey

**Keywords:** *Geotrichum clavatum*, Invasive fungal infection, Multiple myeloma, *Saprochaete clavata*

## Abstract

**Background and Purpose:**

Invasive fungal infections (IFI) are life-threatening and can be seen in immuno-compromised patients with malignancy, those who undergo chemotherapy, or transplant recipients. The *Candida* and *Aspergillus species* are the most common IFI agents; however, infections can also be caused by rare fungal species. This case report is about a bloodstream infection due to *Saprochaete clavata* (formerly known as *Geotrichum clavatum*) in a woman with multiple myeloma.

**Case report:**

A 59-years-old woman suffered from fever, widespread rashes, and diarrhea after an autologous bone marrow transplantation. Peripheral blood cultures were taken from the patient and sent to the microbiology laboratory. Cultures grew white to cream-colored cottony colonies. Moreover, septate and branched hyphae and arthroconidia were seen under a microscope by lactophenol blue staining. The fungi colonies were identified by Maldi Biotyper 3. 1. (manufactured by Bruker Daltonics, USA) as S. clavata (G. clavatum) with a reliable score. Antifungal susceptibility test was carried out by the concentration gradient strip Etest method. Minimal inhibitory concentrations of Amphotericin B, fluconazole, voriconazole, posaconazole, and anidulafungin were determined as 4, 3, 0.125, 0.125, and &gt; 32 mg/dL, respectively. Despite amphotericin B treatment, the patient died three days after the identification of the fungi.

**Conclusion:**

The IFIs are serious conditions that have high mortality rates. In the current case report, we aimed to draw attention to S. clavata which is a rare fungal agent.

## Introduction

Saprochaete (formerly known as Geotrichum) species are arthroconidial yeasts belonging to the Endomycetales family of Ascomycota phylum. The *Saprochaete species* are referred to as yeast-like fungi since their colonies are similar to yeast; however, they produce hyphae-like mold. Some species of * Saprochaete* are parts of human respiratory, intestinal, and skin microbiota; therefore, they have been isolated from human sputum and faeces [ 1 ].  

*Saprochaete species* may cause opportunistic infections in immuno-compromised hosts which are referred to as geotrichosis. *Unlike the* infections *caused by aspergillus and Candida,* *Saprochaete species* infections are uncommon and have been exclusively reported in immuno-compromised or hematological malignancy patients.* Saprochaete* capitate and *Saprochaete clavata* are the most frequently identified* Saprochaete*species in humans [ 2 - 5 ]. 

*Saprochaete species* have fairly typical hyphae and arthroconidia which are barrel-shaped and rectangular or rounded at the ends. Optimal growth temperature of *Saprochaete species* is 25°C, and most strains either do not grow at all or grow weakly at 37°C [ 1 ]. A bloodstream infection caused by *S. clavata* after autologous bone marrow transplantation (ABMT) in a patient with Multiple Myeloma is reported in this study. 

## Case report

A 59-year-old female with multiple myeloma was admitted to the Department Of Internal Medicine at Dicle University Hospital for ABMT. The BMT unit was equipped with HEPA system. The patient had fever episodes after ABMT and empirical therapy with meropenem and vancomycin was started according to the Infectious Diseases Society of America recommendations for high-risk patients which required hospitalization [ 6 ].

The patient also suffered from generalized petechiae, abundant diarrhea, orbital erythema, and vision loss in her left eye. It should be noted that the eye symptoms began the day after hospitalization. Orbital examination revealed conjunctival hemorrhage, orbital edema, and necrosis in the nasal area. Moreover, there was no growth in nasal tissue culture. Results of abdominal computerized tomography revealed diffuse edema in the abdomen. Fluconazole and metronidazole were added to treatment for persistent fever, considering anaerobic or yeast infection. 

It is noteworthy that the patient had diabetes mellitus for 20 years. The laboratory tests revealed pancytopenia; besides, white blood cell, red blood cell, platelet, and hemoglobin counts were 120/Μl (4,600-10,200), 2790/µl (4,040- 6,130), 35040/µl (142,000-424,000), and 5.58 g/Dl (12.2-18.1), respectively. It should be mentioned that neutropenia continued until the death of the patient. In addition, C-reactive protein, sedimentation, and procalcitonin levels were high as 28.7mg/dl (0-0,5), 70 mm/h (0-20), and 40 ng/ml (0-0, 12), respectively. 

After persistent fever episodes, fluconazole was switched to anidulafungin. Blood samples were cultured in BACTEC Plus Aerobic/F bottles and processed by the BACTEC FX (manufactured by Becton Dickinson, USA) automated system. Subcultures were performed on 5% sheep blood agar (SBA), and Sabouraud dextrose agar (SDA) media after positive signaling and microscopic examination of the BACTEC bottles. White to cream cottony colonies grew after 24 h of incubation on SBA and SDA. Gram staining of the colonies revealed septate and branched hyphae and arthroconidia (Figure 1 A, B, C). 

**Figure 1A:**
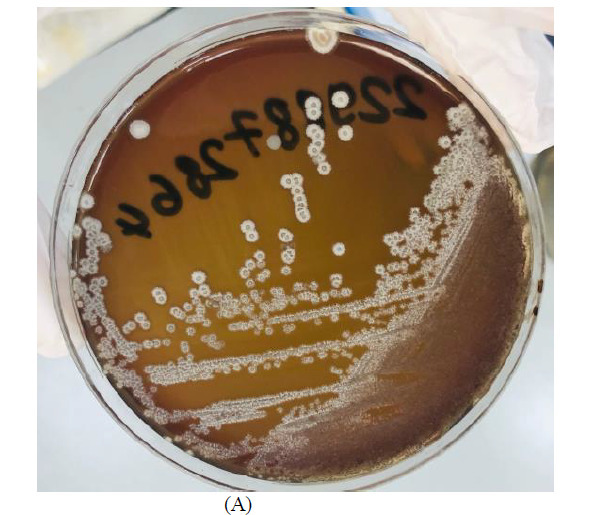
A. *S. clavata* colonies on Sheep Blood Agar

**Figure 1B:**
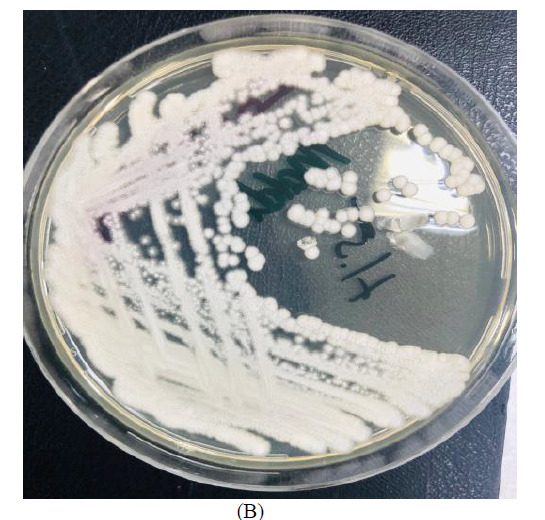
B. *S. clavata* colonies on Sabouraud Dextrose Agar

**Figure 1C:**
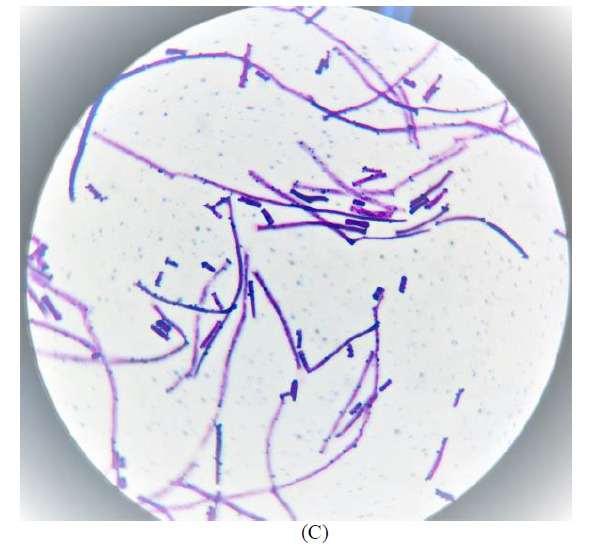
C. Gram staining of *S. clavata* hyphae and arthroconidiae

The isolate was identified as *S. clavata* (*G. clavatum*) by MALDI Biotyper 3.1 (manufactured by Bruker Daltonics, USA) with a score of 2.34 (≥2 scores are reliable at species level). Anidulafungin (50mg/day) was switched to liposomal amphotericin B(300mg/day) after fungal identification. Indirect tests of mannan, beta-D-glucan, galactomannan could not be performed for IFI monitoring as they were not available in our laboratory.

Antifungal susceptibility test (AFST) of the isolate was performed by gradient test method using E-test strips (manufactured by Hi Media, India) on Roswell Park Memorial Institute agar. Minimum inhibitory concentrations (MIC) of amphotericin B, fluconazole, voriconazole, posaconazole, and anidulafungin were found to be 4, 3, 0.125, 0.125, and >32 mg/dl, respectively (Figure 1 D). The patient died of sepsis and multiple organ failure before the determination of the sensitivity test results. 

**Figure 1D:**
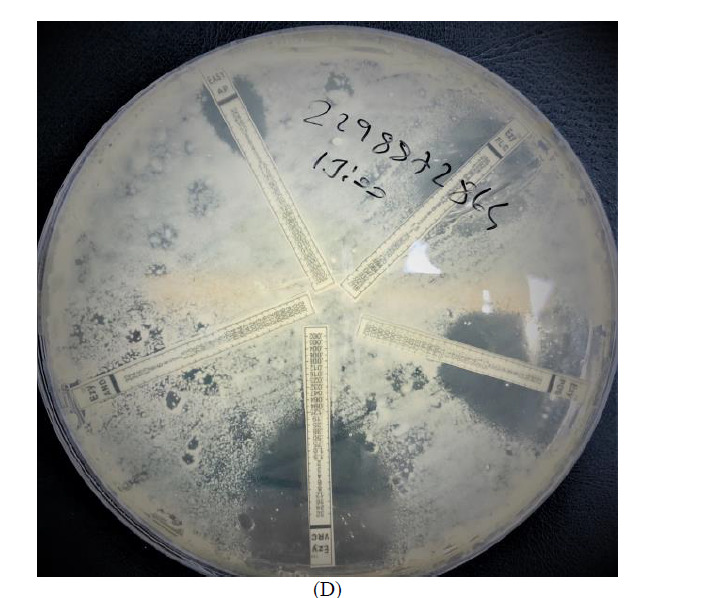
D. Antifungal susceptibility testing of *S. clavata* on RPMI agar medium

## Discussion

Prolonged corticosteroid therapy, immune-suppressive treatment, hematological malignancies, and prolonged broad-spectrum antibiotic therapies have been reported as the risk factors for invasive fungal infections [ 3 , 7 ]. Unlike other reported cases, the patient in our case had multiple myeloma, not leukemia. Due to ABMT and neutropenia, this patient was likely to be susceptible to invasive fungal infections similar to previously reported cases [ 2 , 8 - 10 ]. 

*Candida* and *Aspergillus* species are the most common causes of IFIs, while *Rhodotorula, Trichosporon, Saccharomyces cerevisiae,* and *Geotrichum* species rarely cause IFI [ 7 ]. Among *Geotrichum* species, **G. capitatum** is the most common cause of systemic human infection [ 2 , 3 ]. According to the results of previous studies, during the same period, there were about 100 cases of *G. capitatum* infection which were more than the number of cases infected with *S. clavata* [ 4 , 5 , 8 , 11 , 12 ]. In a study based on the FungiScope™ data during2004-2016 on 505 rare IFI cases,14 and5 *S. capitatum* and *S. clavata* cases infection cases were reported, respectively [ 9 ]. Current number of *S. clavata* cases on the FungiScope™ registry is 12, including two cases in 2017 [ 13 ]. 

In a retrospective study about *S. clavata* infections, Buchta et al. reported colonization and IFI in 48 and 6 patients in the Hemato-Oncology Department of a teaching hospital [ 10 ]. Over the past five years, the number of *S. clavata*-related infections, including FungiScope™ data, has reached about 80 cases [ 4 , 10 , 13 - 15 ]. 

Blood culture is crucial for the diagnosis of IFI caused by *S. clavata* as it allows both identification of isolates and antifungal susceptibility testing. All 12 cases *of the* FungiScope™ registry had blood culture positivity while polymerase chain reaction positivity was reported in only two cases [ 13 ]. Buchta et al. also revealed that the diagnosis of all six patients was based on a positive blood culture [ 10 ]. 

The first *S. clavata* infection was reported in two neutropenic leukemia patients in Italy by Lacroix et al. in 2007 [ 12 ]. Subsequently, Picard et al. reported three fatal cases of *S. clavata* infection in a French hospital. All three cases were acute myeloid leukemia (AML) patients receiving second-line empirical antibiotherapy for febrile neutropenia and antifungal prophylaxis. Similar to our case, the patients died despite antifungal treatments [ 11 ]. The *S. clavata* infection generally occurs in patients with hematological malignancies, such as AML [ 8 , 11 , 12 ]. 

Camus et al. reported the case of another patient with AML who developed *S. clavata* sepsis in France. They found that *S. clavata*, as a member of intestinal microbiota, led to peritonitis followed by sepsis, liver disfunction, and multiorgan failure. They reported that the above-mentioned patient responded to voriconazole treatment [ 8 ]. Our patient suffered from vision loss and retinal hemorrhage, and to our knowledge, there was no evidence of *S. clavata*-related retinitis in the literature. 

Advanced diagnostic techniques, such as mass spectrometry and molecular methods, may have contributed to the increasing number of *S. clavata* infections. Moreover, databases of mass spectrometry systems are crucial in the differentiation of *Saprochaete species*. Vitek-2 and older databases fail to distinguish *S. clavata* from *S. capitatum*, while Maldi Biotyper 3.1 successfully identifies both *Saprochaete species* }[ 9 , 16 , 17 ]. In the current case, the isolate was identified by Maldi Biotyper 3.1. 

Antifungal breakpoint levels have not yet been established in the guidelines as there are not enough studies on *Saprochaete species* [ 18 ]. In a study about an outbreak of *S. clavata* sepsis, Lo Cascio et al. reported that all seven isolates showed high MIC levels to fluconazole and echinocandins and low MIC levels to voriconazole and flucytosine [ 19 ]. 

A report about FungiScope™ registry cases revealed that all five isolates of *S. clavata* showed high (>32 mg/L) MICs to caspofungin [ 9 ]. Previous case reports have indicated that *S. clavata* was resistant to echinocandins and sensitive to voriconazole in [ 2 , 5 , 8 , 10 , 12 ]. In addition, voriconazole and/or liposomal amphotericin B treatments were reported as the most successful ones [ 8 , 9 , 14 , 20 ]. In the current report, *S. clavata* infection developed while the patient was receiving fluconazole treatment. Fluconazole was changed to anidulafungin due to persistent fever episodes; moreover, anidulafungin was switched to amphotericin B after the identification of *S. clavata*. The MICs of all tested medications were found to be far more above the sensitivity limits based on the European Committee on Antimicrobial Susceptibility Testing for common Candida species [ 18 ]. The patient died of sepsis and multiple organ failure just before the conclusion of the susceptibility test was. 

## Conclusion

The IFIs are serious conditions that have high mortality rates. In the current case report, we aimed to draw attention to *S. clavata* which is a rare fungal agent. The *S. clavata* was found to be resistant to the tested antifungals; therefore, the patient died shortly after the infection. However, rapid identification of the yeasts and early initiation of proper treatment can save the lives of IFI patients.

Author’s contribution

M. M. was the supervisor of the research. H. K and N. U performed the laboratory analysis of the samples and contributed to data interpretation. U. M. E performed clinical data interpretation. N. Ö. and H. K wrote the manuscript draft. 

Financial disclosure

No financial support was used for this study.

## Author’s contribution

M. M. was the supervisor of the research. H. K and N. U performed the laboratory analysis of the samples and contributed to data interpretation. U. M. E performed clinical data interpretation. N. Ö. and H. K wrote the manuscript draft.

## Financial disclosure

No financial support was used for this study.

## References

[1] ADDIN Mendeley Bibliography CSL_BIBLIOGRAPHY Larone DH (2011). Medically Important Fungi.

[2] Purohit P ( 2014). Breakthrough disseminated Saprochaete capitata infection in a child with acute myeloid leukaemia receiving caspofungin therapy. JMM Case Reports.

[3] Pemmaraju N ( 2014). , Prieto VG, Jain N, Kontoyiannis DP, Borthakur G. Disseminated Saprochaete capitata (formerly known as Geotrichum capitatum and Blastoschizomyces capitatus) in a patient with acute myeloid leukemia. Eur J Haematol.

[4] Liu X ( 2019). Invasive Fungal Infection Caused by Geotrichum clavatum in a Child with Acute Leukemia: First Documented Case from Mainland China. Jpn J Infect Dis.

[5] Del Principe MI ( 2016). A cluster of Geotrichum clavatum( Saprochaete clavata) infection in haematological patients: a first Italian report and review of literature. Mycoses.

[6] Freifeld AG ( 2011). Clinical practice guideline for the use of antimicrobial agents in neutropenic patients with cancer: 2010 Update by the Infectious Diseases Society of America. Clinical Infectious Diseases.

[7] Chitasombat MN ( 2012). Rare opportunistic( non-Candida, non-Cryptococcus) yeast bloodstream infections in patients with cancer. J Infect.

[8] Camus V ( 2014). Invasive Geotrichum clavatum Fungal Infection in an Acute Myeloid Leukaemia Patient: A Case Report and Review. Mycopathologia.

[9] Durán Graeff L ( 2017). Invasive infections due to Saprochaete and Geotrichum species: Report of 23 cases from the FungiScope Registry. Mycoses.

[10] Buchta V ( 2019). Saprochaete clavata Invasive Infections - A New Threat to Hematological-Oncological Patients. Front Microbiol.

[11] Picard M ( 2014). Concomitant cases of disseminated Geotrichum clavatum infections in patients with acute myeloid leukemia. Leuk Lymphoma.

[12] Lacroix C (2007). Geotrichum clavatum an emerging pathogen responsible for invasive infection in two neutropenic leukemia patients. In: 3rd Trends in Medical Mycology.

[13] FungiScope Query Tool http://www.fungiquest.net/search?_sq=Saprochaete+clavata&_alive=0.

[14] Favre S ( 2016). Saprochaete clavata invasive infection in a patient with severe aplastic anemia: Efficacy of voriconazole and liposomal amphotericin B with adjuvant granulocyte transfusions before neutrophil recovery following allogeneic bone marrow transplantation. Med Mycol Case Rep.

[15] Stanzani M ( 2019). Saprochaete clavata infections in patients undergoing treatment for haematological malignancies: A report of a monocentric outbreak and review of the literature. Mycoses.

[16] Kolecka A ( 2013). Identification of medically relevant species of arthroconidial yeasts by use of matrix-assisted laser desorption ionization-time of flight mass spectrometry. J Clin Microbiol.

[17] Hamprecht A ( 2014). Performance of two MALDI-TOF MS systems for the identification of yeasts isolated from bloodstream infections and cerebrospinal fluids using a time-saving direct transfer protocol. Med Microbiol Immunol.

[18] European Committee on Antimicrobial Susceptibility Testing Antifungal Agents-Breakpoint tables for interpretation of MICs, Version 9.0. 2018. http://www.eucast.org/fileadmin/src/media/PDFs/EUCAST_files/AFST/Clinical_breakpoints/Antifungal_breakpoints_v_9.0_180212.pdf.

[19] Lo Cascio G ( 2020). Outbreak of Saprochaete clavata Sepsis in Hematology Patients: Combined Use of MALDI-TOF and Sequencing Strategy to Identify and Correlate the Episodes. Front Microbiol.

[20] Leoni M ( 2019). Magnusiomyces clavatus infection in a child after allogeneic hematotopoetic stem cell transplantation: Diagnostic and therapeutic implications. Med Mycol Case Rep.

